# Music learning engagement and perceived psychological wellbeing among university students: an indirect association involving perceived emotional intelligence in music learning

**DOI:** 10.3389/fpsyg.2026.1956213

**Published:** 2026-09-08

**Authors:** Honghua Xu, Wei Liu

**Affiliations:** 1 College of Music, Guizhou University, Guiyang, China; 2 Graduate School, José Rizal University, Mandaluyong, Philippines

**Keywords:** Chinese university students, music learning engagement, perceived emotional intelligence in music learning, perceived psychological wellbeing, university music learning

## Abstract

**Objective:**

Music learning involves sustained practice, evaluative feedback, and emotionally demanding experiences, yet limited research has examined how learning engagement and perceived emotion-related competence are jointly associated with psychological wellbeing. This study investigated the associations among music learning engagement, perceived emotional intelligence in music learning, and perceived psychological wellbeing among university students.

**Methods:**

Cross-sectional survey data were collected from 423 students recruited through student networks associated with three universities in Guizhou Province, China, who had participated in formal or organized music-learning activities. Covariance-based structural equation modeling was used to examine the measurement structure and the direct, indirect, and total associations among the study variables. Percentile bootstrap confidence intervals were estimated using 5,000 resamples.

**Results:**

Music learning engagement was positively associated with perceived psychological wellbeing (*β* = 0.419, 95% bootstrap CI [0.305, 0.531]) and perceived emotional intelligence in music learning (*β* = 0.584, 95% bootstrap CI [0.496, 0.665]). Perceived emotional intelligence in music learning was also positively associated with perceived psychological wellbeing after music learning engagement was included in the model (*β* = 0.430, 95% bootstrap CI [0.308, 0.544]). The standardized indirect association was 0.251, 95% bootstrap CI [0.178, 0.326]. The model explained 34.1% of the variance in perceived emotional intelligence in music learning and 57.0% of the variance in perceived psychological wellbeing.

**Conclusion:**

This study provides correlational evidence that music learning engagement, perceived emotional intelligence in music learning, and perceived psychological wellbeing are positively associated among university students. Perceived emotional intelligence in music learning accounted for part of the association between music learning engagement and perceived psychological wellbeing within the specified model. The findings provide a more differentiated understanding of how learning involvement, emotion-related self-perceptions, and psychological functioning are connected in university music-learning contexts.

## Introduction

Psychological wellbeing is an important educational outcome because universities are expected to support not only students’ academic achievement but also their positive functioning during and beyond university life. In this study, perceived psychological wellbeing refers to a broad, study-specific indicator of students’ self-reported positive psychological functioning, including positive life evaluation, optimism, purpose, confidence in managing challenges, and an overall sense of functioning. The measure was informed by flourishing and eudaimonic perspectives (Diener et al., 2010; Ryff, 1989), but it was not intended to reproduce the full multidimensional structure of either framework or to serve as a shortened version of an established wellbeing scale. Reviews have identified academic demands, social relationships, institutional conditions, and access to support as important correlates of university students’ mental health and wellbeing (Campbell et al., 2022; Hyseni Duraku et al., 2024). Empirical studies have further linked supportive educational environments and student engagement with positive psychological functioning (Chaudhry et al., 2024; Zhang, 2024). Evidence from Chinese higher education also suggests that educational support may be associated with psychological wellbeing through students’ motivational and psychological resources (Liu and Zhao, 2026). The Chinese higher education context also warrants specific attention because university students may encounter overlapping academic, economic, and employment-related pressures during the transition toward graduation and adult roles. Recent evidence from Chinese university populations has highlighted the relevance of employment pressure, economic circumstances, academic demands, psychological resources, and social support to students’ mental health and psychological functioning (Fan et al., 2024; Peng et al., 2024). These contextual pressures provide an additional reason to examine how educational engagement and positive psychological functioning are related among Chinese university students. Music learning provides a relevant context for examining these associations because it requires sustained practice, expressive interpretation, responses to evaluative feedback, emotional regulation, and collaboration. Previous studies have associated music-related educational experiences with students’ subjective wellbeing, self-esteem, and psychological development (Fu and Tu, 2023; Jiang, 2024). Other research has highlighted the relevance of music use to learning, wellbeing, emotion-regulation self-efficacy, and related psychological functioning, as well as the potential role of music education in students’ mental health (Hu et al., 2021; Tan et al., 2024; Wang et al., 2022).

However, participation in music learning cannot be assumed to produce uniformly positive outcomes. Music learning may also involve physical strain, repeated frustration, evaluative pressure, and performance-related anxiety, and music students may experience distinctive challenges to health and wellbeing (Alessandri et al., 2020; Ege et al., 2023). Its educational value may therefore depend less on participation alone than on how actively students engage in learning activities (Wang, 2021). Engagement is commonly conceptualized as behavioral, cognitive, and emotional involvement in learning (Fredricks et al., 2004), yet music-related studies have often relied on participation status, attendance, practice frequency, or isolated indicators of involvement. Such indicators may not fully capture the quality of students’ engagement in music learning. Limited attention has also been given to whether students’ perceived emotion-related competence is associated with both music learning engagement and psychological wellbeing. More sustained engagement may provide repeated opportunities to interpret emotional experiences, respond to feedback, manage performance-related difficulties, and coordinate with others. Self-report emotional intelligence concerns individuals’ perceived capacity to understand their own and others’ emotions, regulate emotional responses, and use emotions constructively (Wong and Law, 2002). Emotion-regulation processes are relevant to affective, cognitive, and social functioning, providing a broader basis for examining their association with psychological wellbeing (Gross, 2002). These perceptions have been associated with university students’ motivation, self-efficacy, academic achievement, and psychological wellbeing (Chang and Tsai, 2022; Shengyao et al., 2024). General assessments, however, may not fully correspond to the specific emotional and evaluative situations encountered during music learning. Contextualizing self-report emotional-intelligence items to a particular setting can make the situational referent explicit while retaining the underlying emotion-related domains being assessed. Recent work has similarly emphasized context-sensitive assessment of self-reported emotional intelligence (Audrin and Audrin, 2024), while educational research has developed and validated domain-specific emotional-intelligence measures within particular learning contexts, such as second-language learning (Alamer and Alrabai, 2024). Such evidence supports distinguishing general emotional-intelligence dispositions from students’ self-perceived application of emotion-related capacities within a specific educational domain. The present study therefore uses the term perceived emotional intelligence in music learning to describe students’ self-perceived application of established emotion-related capacities within music-learning activities. This contextualization aligns the item content with the learning setting but does not imply a distinct form of emotional intelligence unique to music learning or equivalence with a validated general trait emotional-intelligence scale. It also differs from music-related emotion regulation, which concerns using music to alter emotional states rather than applying emotion-related competence while learning music.

Accordingly, this study examines the associations among music learning engagement, perceived emotional intelligence in music learning, and perceived psychological wellbeing among university students. It investigates whether perceived emotional intelligence in music learning statistically accounts for part of the association between music learning engagement and perceived psychological wellbeing. The study contributes by assessing music learning engagement through indicators informed by behavioral, cognitive, and emotional engagement perspectives rather than relying solely on participation or exposure. It also examines whether students’ perceived application of emotion-related capacities within music learning is statistically associated with both engagement and broader positive functioning. Given the cross-sectional design, the proposed ordering is theoretically specified and is not interpreted as evidence of temporal or causal mediation.

## Literature review and hypotheses

### Music learning engagement and perceived psychological wellbeing

Psychological wellbeing extends beyond the absence of psychological distress and encompasses positive functioning in areas such as competence, supportive relationships, purpose, optimism, and engagement in meaningful activities (Diener et al., 2010). Self-determination theory offers an interpretive perspective on why fuller involvement in educational activities may be associated with positive functioning. According to the theory, psychological growth and wellbeing are supported by the satisfaction of the basic psychological needs for autonomy, competence, and relatedness (Ryan and Deci, 2000). Student engagement is commonly conceptualized as behavioral, cognitive, and emotional involvement in learning, including participation and persistence, interest and affective connection, and attention and strategic thinking (Fredricks et al., 2004). Although engagement is not equivalent to basic psychological need satisfaction, fuller involvement in learning may coincide with experiences of choice, progress, and constructive social connection. Engagement may therefore be positively associated with psychological wellbeing. In the present study, however, SDT serves only as a broad interpretive framework rather than as a mechanism directly evaluated by the empirical model. Basic psychological need satisfaction was not measured, and the proposed associations should therefore not be interpreted as a direct test of the need-satisfaction processes specified by SDT.

Music learning provides a relevant context for examining this association because it involves sustained practice, expressive interpretation, reflection, performance, and interaction with instructors or peers. Greater engagement may coincide with experiences of accomplishment, meaningful self-expression, and social connection. Existing studies have linked music-related learning experiences and participation in group music activities with subjective wellbeing, self-esteem, personal development, positive social connections, and other indicators of adaptive functioning (Alessandri et al., 2020; Fu and Tu, 2023; Jiang, 2024; Yang et al., 2025). However, these studies have used varied indicators of participation and engagement. Because music learning may also involve evaluative pressure, repetitive practice, and performance demands, the quality of students’ behavioral, cognitive, and emotional involvement may be more informative than enrollment, attendance, or exposure alone.

Based on these theoretical arguments and empirical findings, the following hypothesis is proposed:

*H1*: Music learning engagement is positively associated with perceived psychological wellbeing.

### Music learning engagement and perceived emotional intelligence in music learning

Perceived emotional intelligence in music learning refers to students’ self-perceived capacity to understand their own and others’ emotions, regulate emotional responses, and use emotions constructively during music-learning activities (Wong and Law, 2002). It is treated as a contextualized perception of emotional competence rather than an objective ability or a distinct form of emotional intelligence.

Music learning involves emotionally demanding activities, including interpreting musical expression, responding to evaluative feedback, managing frustration during practice, and regulating tension during performance. These demands make emotion-related competence particularly relevant because students must recognize emotional responses, interpret their sources, regulate frustration or tension, and use emotional information constructively while remaining engaged with learning tasks. Broader evidence also indicates that active engagement in musical activities is related to emotion-regulation processes (Peters et al., 2024). More directly, research among Chinese university music students has shown that higher emotional intelligence is associated with lower music performance anxiety, suggesting that emotion-related competence may represent a potentially protective psychological resource in evaluative performance contexts (Zhang and Jenatabadi, 2024). However, this evidence does not establish a buffering or moderating effect, and the present study likewise does not test whether perceived emotional intelligence attenuates the effects of performance pressure or other musical stressors. The proposed ordering treats music learning engagement as the more proximal educational-context variable because it reflects students’ ongoing participation, persistence, cognitive investment, and affective involvement. Repeated engagement may expose students to situations in which these emotion-related capacities become salient. Perceived emotional intelligence in music learning is therefore positioned as a possible correlate within the statistical association between engagement and broader psychological functioning. The ordering is theoretically motivated rather than empirically established, and the reverse possibility that students with stronger perceived emotion-related competence engage more actively remains plausible. Accordingly, the following hypothesis is proposed:

*H2*: Music learning engagement is positively associated with perceived emotional intelligence in music learning.

### Perceived emotional intelligence in music learning and perceived psychological wellbeing

Students’ perceptions of their emotion-related competence may be relevant to psychological wellbeing because they are associated with how students interpret and manage emotionally demanding learning experiences. From a flourishing perspective, psychological wellbeing involves positive functioning, purposeful engagement, supportive relationships, and a sense of competence in everyday life (Diener et al., 2010). Students who perceive themselves as emotionally competent during music learning may therefore also evaluate their broader psychological functioning more positively when facing the demands of practice, feedback, and performance. This reasoning is consistent with broader evidence linking individual differences in emotion-regulation processes to affective functioning, relationships, and wellbeing (Gross and John, 2003).

A recent study of Chinese university students reported a positive association between general emotional intelligence and psychological wellbeing (Shengyao et al., 2024). Although that study did not examine context-specific perceptions of emotional competence, it suggests that students who perceive themselves as better able to understand and regulate emotions also tend to evaluate their psychological functioning more positively. Accordingly, the following hypothesis is proposed:

*H3*: Perceived emotional intelligence in music learning is positively associated with perceived psychological wellbeing.

### The indirect association involving perceived emotional intelligence in music learning

Taken together, the preceding arguments motivate examination of whether the covariance between music learning engagement and perceived psychological wellbeing can be statistically decomposed into a direct component and an indirect component involving perceived emotional intelligence in music learning. This specification represents one theoretically motivated ordering of the observed associations rather than a validated intermediate psychological mechanism. Because all three constructs were measured concurrently, alternative directional orderings remain plausible and cannot be distinguished temporally in the present design. Accordingly, the following hypothesis is proposed:

*H4*: Within the theoretically specified cross-sectional model, music learning engagement shows a statistically distinguishable indirect association with perceived psychological wellbeing involving perceived emotional intelligence in music learning.

**Figure 1:**
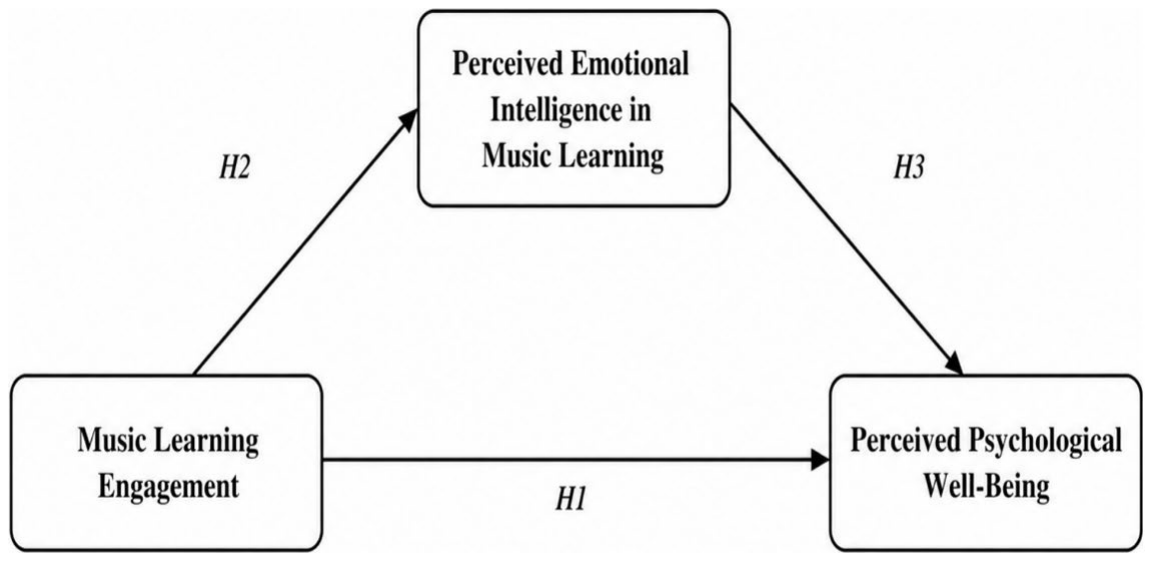
Conceptual framework of the proposed research model. H1–H3 represent the hypothesized direct associations among the study variables, and H4 represents the hypothesized indirect association. The directional arrows indicate the theoretically specified ordering tested in the cross-sectional model and should not be interpreted as evidence of temporal or causal direction.

Taken together, H1–H4 form the proposed conceptual model shown in Figure 1. The model specifies direct associations of music learning engagement with perceived psychological wellbeing and perceived emotional intelligence in music learning, as well as an indirect association between music learning engagement and perceived psychological wellbeing involving perceived emotional intelligence in music learning.

## Method

### Participants and procedure

This study used a cross-sectional survey design to examine the associations among music learning engagement, perceived emotional intelligence in music learning, and perceived psychological wellbeing among university students. Participants were recruited through student networks at three universities in Guizhou Province, China, using convenience sampling. Eligible participants were currently enrolled students who had taken part in at least one formal university music course, music-related elective, or organized music-learning activity during the current academic year. This eligibility criterion encompassed heterogeneous forms of music learning that could differ in instructional formality, intensity, duration, disciplinary relevance, and performance exposure. The sample should therefore not be interpreted as consisting exclusively of music majors or students receiving intensive professional music training. Detailed information on music-major status, prior music training, practice frequency, type and intensity of music-learning activity, curricular or extracurricular participation, and performance exposure was not collected. This heterogeneity should therefore be considered when interpreting and generalizing the findings. Because institutional affiliation was not retained, the number of participants from each university could not be determined. Data were collected through Wenjuanxing from February 23 to April 7, 2026. Before participation, students received information about the study purpose, the voluntary nature of participation, data confidentiality and anonymity, and their right to withdraw before submitting the questionnaire. Electronic informed consent was obtained from all participants. No personally identifiable information was collected, and no financial or academic incentives were provided.

A total of 423 complete questionnaires were submitted and included in the analysis. Because all questionnaire items were required, no item-level missing data were present. The final sample included 201 male students (47.5%) and 222 female students (52.5%). Participant characteristics are presented in Table 1.

**Table 1:** Demographic characteristics of the participants.

Characteristic	Category	*n*	%
Gender	Male	201	47.5
	Female	222	52.5
Grade	Freshman	113	26.7
	Sophomore	88	20.8
	Junior	103	24.3
	Senior	119	28.1
Field of study	Arts	178	42.1
	Humanities and Social Sciences	132	31.2
	Science and Engineering	113	26.7

*n* = 423.

### Measures

All constructs were assessed using five items rated on a five-point Likert scale ranging from 1 (strongly disagree) to 5 (strongly agree), with higher scores indicating higher levels of the corresponding construct. Item development was informed by established theoretical frameworks and existing measures. The music learning engagement and perceived emotional intelligence items were contextualized to university music learning, whereas the perceived psychological wellbeing items assessed broader positive psychological functioning.

Two experts with backgrounds in educational psychology and music education reviewed the initial item pool for conceptual relevance, clarity, and contextual appropriateness. The items were subsequently pilot-tested with 30 university students who were not included in the main sample. The pilot was used to assess item comprehensibility, wording, and contextual appropriateness rather than to conduct factor-analytic item selection, for which the pilot sample was not intended. Minor wording revisions were made before formal data collection. The item set was therefore finalized before analysis of the main sample, and no items were selected, deleted, or modified *post hoc* on the basis of the measurement or structural results obtained from the main study. These procedures provided preliminary support for the content relevance, contextual appropriateness, and comprehensibility of the items. Because the three study-specific measures were evaluated in the same main sample subsequently used to estimate the structural associations, the evidence obtained in the present study should be regarded as preliminary and sample-specific rather than as independent psychometric validation.

#### Music learning engagement

Music learning engagement was assessed using five context-specific items informed by the behavioral, cognitive, and emotional engagement framework proposed by Fredricks et al. (2004). The items assessed active participation in music-learning activities, effort to understand musical knowledge, positive involvement in music learning, persistence when encountering learning difficulties, and reflection on learning experiences. A sample item was, “I actively participate in music-learning activities”.

The five items were modeled as indicators of a broad overall engagement construct. Although the item content reflected behavioral, cognitive, and emotional aspects of engagement, the measure was not designed to estimate these aspects as separate subscales.

#### Perceived emotional intelligence in music learning

Perceived emotional intelligence in music learning was assessed using five context-specific items developed with reference to the conceptual domains of the Wong and Law Emotional Intelligence Scale (WLEIS; Wong and Law, 2002), including self-emotional appraisal, others’ emotional appraisal, regulation of emotion, and use of emotion. The items assessed students’ perceived capacity to recognize and understand their own emotions, understand the emotions of others, regulate emotional responses, and use emotions constructively during music-learning activities. A sample item was, “I can regulate my emotions when I face challenges in music learning”.

The items were contextualized to music-learning activities and modeled as indicators of a single overall construct. The measure assessed students’ self-perceived application of emotion-related capacities within music learning rather than objectively measured emotional ability.

#### Perceived psychological wellbeing

Perceived psychological wellbeing was assessed using five items developed with reference to broad accounts of positive psychological functioning, including the flourishing perspective of Diener et al. (2010) and the eudaimonic perspective of Ryff (1989). The items assessed positive life evaluation, optimism about the future, perceived purpose, confidence in managing challenges, and an overall sense of positive psychological functioning. A sample item was, “I feel optimistic about my future”.

The five items were modeled as indicators of a broad overall construct of perceived psychological wellbeing. The measure was intended to capture students’ general perceptions of positive psychological functioning rather than reproduce the complete multidimensional structure of either the Flourishing Scale or Ryff’s model of psychological wellbeing. It should therefore be interpreted as a study-specific indicator of broad perceived positive psychological functioning rather than as a shortened version of either established framework.

### Data analysis

Descriptive statistics, missing-data screening, and univariate normality assessments were conducted in SPSS 26.0, while confirmatory factor analysis and covariance-based structural equation modeling were performed in AMOS 28.0. Multivariate normality was assessed using Mardia’s statistics. Multivariate skewness was not statistically significant, χ^2^ (680) = 668.43, *p* = 0.617, whereas normalized multivariate kurtosis differed significantly from zero, *z* = −3.27, *p* = 0.001. Given the limited univariate departures from normality and the sample size, maximum likelihood estimation was retained, and percentile bootstrap confidence intervals based on 5,000 resamples were used for inference concerning direct, indirect, and total associations. Potential common method variance was examined using Harman’s single-factor test and confirmatory factor model comparisons, with the hypothesized three-factor measurement model compared against a one-factor model and theoretically plausible two-factor alternatives. Consistent with recommendations for research relying on single-source self-report data, these analyses were treated as diagnostic checks rather than as definitive evidence that common method bias was absent (Podsakoff et al., 2003). The measurement model was evaluated using standardized factor loadings, Cronbach’s alpha, composite reliability, average variance extracted, the Fornell–Larcker criterion (Fornell and Larcker, 1981), and heterotrait-monotrait ratios of correlations. Because AMOS does not directly provide HTMT estimates, the HTMT ratios were calculated in Python from the item-level Pearson correlation matrix following Henseler et al. (2015). Model fit was assessed using χ^2^, CFI, TLI, RMSEA, and SRMR, with evaluation based on the overall pattern of indices rather than strict reliance on a single cutoff value (Hu and Bentler, 1999; Kline, 2023). After evaluation of the measurement model, a theoretically ordered structural model was estimated to examine the direct and indirect associations among music learning engagement, perceived emotional intelligence in music learning, and perceived psychological wellbeing. An indirect association was considered statistically distinguishable from zero when its 95% percentile bootstrap confidence interval excluded zero. Because all constructs were measured concurrently, the proposed ordering was theoretically specified rather than temporally established, and the indirect association was interpreted as a model-dependent decomposition of covariance rather than evidence of causal mediation. Because the structural relations among the three latent variables were just-identified, the structural model reproduced the same global fit indices as the measurement model. Explained variance in perceived emotional intelligence in music learning and perceived psychological wellbeing was reported using squared multiple correlations (R^2^).

## Results

### Descriptive statistics and internal consistency

Descriptive statistics and internal consistency estimates for the three study constructs are presented in Table 2. No missing values were identified in the analysis dataset (*n* = 423). Construct-level scores showed no substantial departures from univariate normality, with absolute skewness values ranging from 0.133 to 0.234 and absolute excess kurtosis values ranging from 0.201 to 0.639. All three constructs demonstrated satisfactory internal consistency, with Cronbach’s alpha coefficients ranging from 0.828 to 0.871.

**Table 2:** Descriptive statistics and internal consistency estimates.

Construct	Items	*M*	*SD*	Skewness	Kurtosis	*α*
Music learning engagement	5	3.809	0.632	−0.133	−0.639	0.871
Perceived emotional intelligence in music learning	5	3.782	0.576	−0.224	−0.201	0.833
Perceived psychological wellbeing	5	3.850	0.572	−0.234	−0.475	0.828

*M* = mean; SD = standard deviation; *α* = Cronbach’s alpha.

### Common method diagnostics and competing measurement models

Potential common method variance was examined using Harman’s single-factor test and confirmatory factor model comparisons. In the unrotated exploratory factor analysis, the first factor accounted for 42.92% of the total item variance. This result was treated as a descriptive diagnostic rather than as evidence that common method variance was absent.

The hypothesized three-factor measurement model was subsequently compared with a one-factor model and three theoretically plausible two-factor alternatives. As shown in Table 3, the three-factor model fitted the data substantially better than the one-factor and two-factor models. The one-factor model demonstrated poor fit, χ^2^ (90) = 644.72, CFI = 0.793, TLI = 0.758, RMSEA = 0.121, and SRMR = 0.084. The three-factor model demonstrated substantially better fit than the one-factor model and each of the two-factor alternatives across CFI, TLI, RMSEA, and SRMR. The corresponding likelihood-ratio comparisons were also statistically significant, all *p* < 0.001.

**Table 3:** Comparison of competing measurement models.

Model	χ^2^	df	CFI	TLI	RMSEA	SRMR	Comparison with three-factor model
Three-factor model	94.66	87	0.997	0.997	0.014	0.028	–
Two-factor: MLE and EI combined	465.52	89	0.859	0.834	0.100	0.075	Δχ^2^ (2) = 370.86, *p* < 0.001
Two-factor: MLE and PWB combined	370.52	89	0.895	0.876	0.087	0.064	Δχ^2^ (2) = 275.86, p < 0.001
Two-factor: EI and PWB combined	316.16	89	0.915	0.900	0.078	0.057	Δχ^2^ (2) = 221.50, p < 0.001
One-factor model	644.72	90	0.793	0.758	0.121	0.084	Δχ^2^ (3) = 550.06, p < 0.001

MLE, music learning engagement; EI, perceived emotional intelligence in music learning; PWB, perceived psychological wellbeing. In each two-factor model, the two named constructs were combined into one factor, while the remaining construct was modeled separately.

These comparisons supported the empirical distinction among music learning engagement, perceived emotional intelligence in music learning, and perceived psychological wellbeing, and indicated that a single undifferentiated factor did not adequately reproduce the item covariance structure. They did not, however, rule out method-related inflation because all constructs were assessed through self-report at a single time point.

### Measurement model and construct validity

The retained three-factor measurement model demonstrated very good fit, χ^2^ (87) = 94.66, *p* = 0.269, χ^2^/df = 1.09, CFI = 0.997, TLI = 0.997, RMSEA = 0.014, and SRMR = 0.028.

As shown in Table 4, all standardized factor loadings were statistically significant at *p* < 0.001 and ranged from 0.652 to 0.775. Composite reliability exceeded 0.80 for all three constructs. Music learning engagement met the conventional AVE benchmark, with an AVE of 0.575. The AVE estimates for perceived emotional intelligence in music learning (0.499) and perceived psychological wellbeing (0.491) were marginally below 0.50. Both constructs nevertheless demonstrated composite reliability above 0.80, statistically significant standardized loadings, and no loading below 0.65. The results therefore supported satisfactory internal consistency but only borderline convergent validity for these two constructs.

**Table 4:** Standardized factor loadings and convergent validity.

Construct	Indicator	Standardized loading	CR	AVE
Music learning engagement	MLE1	0.775	0.871	0.575
	MLE2	0.754		
	MLE3	0.769		
	MLE4	0.750		
	MLE5	0.745		
Perceived emotional intelligence in music learning	EI1	0.699	0.833	0.499
	EI2	0.705		
	EI3	0.723		
	EI4	0.711		
	EI5	0.695		
Perceived psychological wellbeing	PWB1	0.717	0.828	0.491
	PWB2	0.707		
	PWB3	0.693		
	PWB4	0.652		
	PWB5	0.731		

All standardized factor loadings were statistically significant at *p* < 0.001. CR, composite reliability; AVE, average variance extracted.

Discriminant validity was assessed using the Fornell–Larcker criterion and heterotrait-monotrait ratios of correlations. As presented in Table 5, the square root of the AVE for each construct exceeded its correlations with the other constructs, although the margin between perceived emotional intelligence in music learning and perceived psychological wellbeing was relatively narrow. The HTMT value for these two constructs was 0.673, below the conservative threshold of 0.85. The remaining HTMT values were also below 0.85, supporting the empirical distinction among the three constructs within the present sample.

**Table 5:** Discriminant validity assessment.

*Panel A. Fornell-Larcker Criterion*			
Construct	1	2	3
1. Music learning engagement	**0.759**		
2. Perceived emotional intelligence in music learning	0.584	**0.707**	
3. Perceived psychological wellbeing	0.670	0.674	**0.700**

*Panel B. HTMT Ratios*	
Construct pair	HTMT
Music learning engagement and perceived emotional intelligence in music learning	0.583
Music learning engagement and perceived psychological wellbeing	0.672
Perceived emotional intelligence in music learning and perceived psychological wellbeing	0.673

Bold diagonal values are the square roots of AVE. Values below the diagonal are latent correlations. HTMT, heterotrait-monotrait ratio of correlations.

### Structural associations

**Figure 2:**
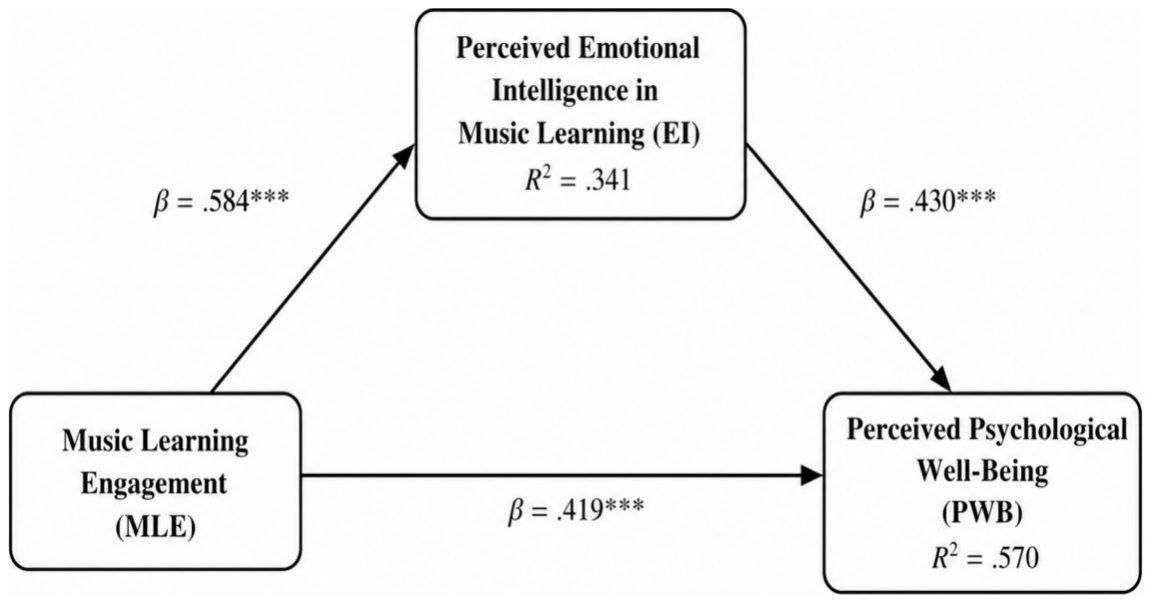
The theoretically ordered structural model with standardized estimates. Values on the arrows represent standardized path coefficients. *R*^2^ values indicate the proportion of variance explained in endogenous constructs. ^***^p < 0.001.

Because the structural relations among the three latent variables were just-identified, the structural model reproduced the same global fit indices as the measurement model. These fit indices therefore reflected the adequacy of the measurement structure rather than the superiority of the specified directional ordering over alternative plausible orderings. The standardized structural coefficients are presented in Table 6 and illustrated in Figure 2.

**Table 6:** Structural path estimates.

Relation	Path	*β*	Bootstrap SE	95% percentile CI	*p*
H1	MLE → PWB	0.419	0.058	[0.305, 0.531]	<0.001
H2	MLE → EI	0.584	0.043	[0.496, 0.665]	<0.001
H3	EI → PWB	0.430	0.059	[0.308, 0.544]	<0.001

Bootstrap standard errors and percentile confidence intervals were estimated using 5,000 resamples. *β*, standardized path coefficient; MLE, music learning engagement; EI, perceived emotional intelligence in music learning; PWB, perceived psychological wellbeing.

Music learning engagement was positively associated with perceived psychological wellbeing, *β* = 0.419, 95% percentile bootstrap CI [0.305, 0.531], consistent with H1. Music learning engagement was also positively associated with perceived emotional intelligence in music learning, *β* = 0.584, 95% percentile bootstrap CI [0.496, 0.665], consistent with H2. Perceived emotional intelligence in music learning was positively associated with perceived psychological wellbeing after music learning engagement was included in the model, *β* = 0.430, 95% percentile bootstrap CI [0.308, 0.544], consistent with H3.

Music learning engagement explained 34.1% of the latent variance in perceived emotional intelligence in music learning. Music learning engagement and perceived emotional intelligence in music learning jointly explained 57.0% of the latent variance in perceived psychological wellbeing.

### Indirect and total associations

The standardized indirect association between music learning engagement and perceived psychological wellbeing involving perceived emotional intelligence in music learning was 0.251, with a 95% percentile bootstrap confidence interval of [0.178, 0.326]. Because the confidence interval excluded zero, the indirect association was statistically distinguishable from zero, consistent with H4. The direct association between music learning engagement and perceived psychological wellbeing also remained statistically distinguishable from zero after perceived emotional intelligence in music learning was included in the model.

The standardized total association was 0.670, with a 95% percentile bootstrap confidence interval of [0.591, 0.740] (Table 7). Taken together, the estimates indicated that perceived emotional intelligence in music learning accounted statistically for part of the association between music learning engagement and perceived psychological wellbeing within the theoretically specified cross-sectional model. This pattern represents a model-dependent decomposition of covariance and does not establish temporal or causal mediation.

**Table 7:** Bootstrap decomposition of direct, indirect, and total associations.

Association	Standardized estimate	Bootstrap SE	95% percentile CI	*p*
Direct association	0.419	0.058	[0.305, 0.531]	<0.001
Indirect association	0.251	0.038	[0.178, 0.326]	<0.001
Total association	0.670	0.038	[0.591, 0.740]	<0.001

Confidence intervals were estimated using 5,000 nonparametric percentile bootstrap resamples. The indirect estimate represents a model-dependent decomposition of covariance within the theoretically specified cross-sectional ordering and should not be interpreted as evidence of temporal mediation.

## Discussion

This study examined the direct and indirect associations among music learning engagement, perceived emotional intelligence in music learning, and perceived psychological wellbeing among university students. The findings supported the hypothesized pattern of associations.

### Music learning engagement and perceived psychological wellbeing

Music learning engagement was positively associated with perceived psychological wellbeing. This finding is broadly consistent with research linking student engagement to adaptive educational outcomes and music-related learning experiences to positive psychological functioning (Fredricks et al., 2004; Fu and Tu, 2023; Jiang, 2024). Students reporting greater engagement in music learning also tended to evaluate their broader psychological functioning more positively. Fuller involvement in music learning may coincide with experiences of accomplishment, progress, and meaningful participation. Sustained practice may allow students to observe the development of their knowledge and skills, while interpretive and performance activities may provide opportunities for self-expression and goal pursuit. These experiences are relevant to the broad conception of perceived psychological wellbeing examined in the present study.

The findings provide context-specific evidence of this association in university music learning, where students often coordinate technical practice with expressive and interpretive demands. Nevertheless, the observed association does not establish that engagement improves perceived psychological wellbeing. Students with stronger perceived psychological wellbeing may be more willing or able to engage actively, while previous musical experience, motivational characteristics, and contextual resources may be associated with both constructs.

### Music learning engagement and perceived emotional intelligence in music learning

Music learning engagement was positively associated with perceived emotional intelligence in music learning. Students reporting greater involvement in their music studies also tended to evaluate their emotion-related competence within that context more positively. This finding adds context-specific evidence linking engagement not only to participation and motivation but also to students’ perceptions of how they understand and manage emotions during learning (Wang, 2021). Practice, evaluative feedback, and performance may expose students to frustration, uncertainty, and performance-related tension. Students who engage more fully with these activities may therefore encounter more opportunities to recognize their emotional responses, reflect on their sources, and identify ways of managing them. Adjacent research has associated emotional intelligence with learning motivation, self-efficacy, and academic functioning among university students (Chang and Tsai, 2022), as well as with psychological outcomes in music-education contexts (Wang et al., 2022).

This association should be interpreted in relation to students’ self-perceived emotion-related competence within music learning rather than general or objectively assessed emotional ability. Its direction also remains uncertain. Students who perceive themselves as more emotionally capable may engage more actively, while sustained engagement may increase their familiarity with the emotional demands of music learning.

### Perceived emotional intelligence in music learning, perceived psychological wellbeing, and the indirect association

Perceived emotional intelligence in music learning was positively associated with perceived psychological wellbeing after music learning engagement was included in the model. Students who evaluated their emotion-related competence more positively also tended to report stronger positive psychological functioning. This finding is consistent with broader evidence linking emotional intelligence to psychological wellbeing among university students (Shengyao et al., 2024) and with research indicating that emotional intelligence is relevant to psychological outcomes in music-education contexts (Wang et al., 2022). Within music learning, perceived emotion-related competence may be associated with how students interpret and respond to the demands of practice, feedback, assessment, and performance. Students who perceive themselves as better able to understand and regulate their emotions may also evaluate their confidence, purpose, optimism, and broader functioning more positively. Part of this association may also reflect shared positive self-evaluation because both constructs were assessed through self-report.

The statistically distinguishable indirect association indicated that perceived emotional intelligence in music learning accounted statistically for part of the association between music learning engagement and perceived psychological wellbeing within the specified model. This pattern is broadly compatible with research linking academic engagement to student wellbeing in higher education (Chaudhry et al., 2024; Zhang, 2024) and with adjacent evidence associating music learning with psychological development and wellbeing-related outcomes (Fu and Tu, 2023; Jiang, 2024). However, because all three constructs were assessed using positively framed self-report items in the same survey, shared positive self-evaluation and evaluative consistency may have contributed to the magnitude of the observed indirect association. The indirect estimate therefore represents a model-dependent decomposition of covariance and does not establish a temporal, causal, or intermediate psychological mechanism.

### Theoretical implications

The main theoretical value of the study lies in differentiating three conceptually related but distinct constructs within university music learning. Music learning engagement represents students’ involvement in learning activities, perceived emotional intelligence in music learning represents their self-evaluated emotion-related competence within those activities, and perceived psychological wellbeing represents broader positive functioning beyond the immediate learning context. Distinguishing these constructs helps avoid treating learning involvement, perceived emotional competence, and broader positive functioning as interchangeable indicators of a single favorable student disposition. The competing measurement models and discriminant-validity analyses provided preliminary, sample-specific support for this distinction. However, because the three study-specific measures were evaluated in the same sample used to estimate the structural associations, these findings should not be interpreted as independent validation of the instruments. The borderline convergent validity of perceived emotional intelligence in music learning and perceived psychological wellbeing further warrants caution when interpreting the structural coefficients.

The study also provides a context-sensitive account of perceived emotional intelligence by examining emotion-related self-perceptions in relation to practice, evaluative feedback, performance demands, and emotional expression. This contextualization does not establish a form of emotional intelligence unique to music learning. Instead, it strengthens the correspondence between the construct and the educational activities in which students evaluate their emotional competence. Self-determination theory offers one possible perspective for interpreting the association between music learning engagement and perceived psychological wellbeing, but autonomy, competence, relatedness, and basic psychological need satisfaction were not directly measured. The findings should therefore not be interpreted as a direct test of the motivational mechanisms specified by the theory.

### Practical implications

The present findings do not establish the effectiveness of any instructional intervention, but they identify educational features that warrant further investigation. In university music-learning settings, educators may consider students’ persistence, reflection, responses to feedback, and perceived ability to manage emotionally demanding learning experiences when examining patterns of engagement and psychological functioning. These associations should not be interpreted as evidence that increasing engagement or strengthening emotion-related competence will improve psychological wellbeing. Rather, they identify potentially relevant educational conditions for future longitudinal and intervention-based research.

### Limitations and future research

Several limitations should be considered when interpreting the findings. The cross-sectional design did not establish the temporal ordering of music learning engagement, perceived emotional intelligence in music learning, and perceived psychological wellbeing. Because the three-variable structural model was just-identified, alternative directional orderings would reproduce equivalent global fit and therefore could not be distinguished statistically using the present cross-sectional data. The indirect association should therefore be interpreted as a model-dependent decomposition of covariance rather than evidence of a causal or developmental process. All variables were assessed using positively framed self-report items in the same survey, so shared response tendencies, social desirability, evaluative consistency, and general positive self-perception may have inflated the observed associations and the proportion of variance explained in perceived psychological wellbeing. Future research should use longitudinal, repeated-measures, or intervention-based designs and incorporate behavioral, performance-based, or observer-reported evidence.

An additional important limitation concerns the measurement foundation of the study. The three study-specific measures were evaluated in the same sample used to estimate the structural model and therefore require independent validation. Consequently, the observed measurement structure and structural associations may partly reflect sample-specific covariance patterns, and the present analyses should be regarded as preliminary construct validation rather than independent confirmation of the measures. Although expert review, pilot testing, confirmatory factor analysis, and reliability assessment provided preliminary support, the AVE estimates for perceived emotional intelligence in music learning and perceived psychological wellbeing were marginally below 0.50. The psychological wellbeing measure was also broad, and its item concerning confidence in managing challenges was conceptually adjacent to self-efficacy, which may have contributed to overlap with other favorable self-evaluations. Future independent validation studies should therefore examine whether item wording and construct coverage can be refined to reduce residual measurement error and improve convergent validity without sacrificing the intended conceptual breadth of the constructs. In addition, the eligibility criterion encompassed heterogeneous forms of music learning, while detailed information on music-major status, prior music training, practice frequency, activity type and intensity, curricular or extracurricular participation, and performance exposure was not collected. Music learning engagement may therefore not represent an identical educational experience across all participants, and the findings should not be generalized to a single uniform form of university music education or specifically to professionally trained music students. Convenience sampling through student networks associated with three universities in Guizhou Province also limits representativeness and generalizability. Recruitment through such networks may have disproportionately reached students who were more socially connected, more interested in music-learning activities, or more willing to participate. Although the sample size was adequate for the planned SEM analyses, this does not eliminate potential self-selection bias or establish representativeness. Because institutional affiliation was not retained, potential clustering of students within universities could not be evaluated. If students from the same university shared similar instructional environments, institutional resources, or music-learning opportunities, the independence assumption underlying the single-level analysis may have been imperfect. Consequently, the standard errors reported for the structural coefficients in Table 6 may have been underestimated, potentially making the corresponding statistical significance tests appear more certain than they would under an analysis accounting for institutional clustering. Future studies should retain institution identifiers, evaluate intraclass correlations, and determine whether cluster-robust standard errors or multilevel modeling are required. They should also use independently validated measures, more diverse samples, and more detailed indicators of students’ music-learning contexts.

## Conclusion

This study examined the direct and indirect associations among music learning engagement, perceived emotional intelligence in music learning, and perceived psychological wellbeing among university students. The findings showed that music learning engagement was positively associated with both perceived emotional intelligence in music learning and perceived psychological wellbeing, while perceived emotional intelligence in music learning was also positively associated with perceived psychological wellbeing. A statistically distinguishable indirect association further indicated that perceived emotional intelligence in music learning accounted for part of the association between music learning engagement and perceived psychological wellbeing within the specified model. The study contributes theoretically by distinguishing learning involvement, context-specific perceptions of emotion-related competence, and broader positive psychological functioning as related but non-interchangeable constructs, thereby providing a more differentiated account of their interrelationships in university music learning. From a practical perspective, the findings point to active participation and support for managing the emotional demands of practice, feedback, assessment, and performance as relevant directions for future educational research. Overall, the study clarifies how music learning engagement, perceived emotional intelligence in music learning, and perceived psychological wellbeing are associated within the university music-learning context.

## Data Availability

The raw data supporting the conclusions of this article will be made available by the authors, without undue reservation.
